# Cognitive psychology: the experience you don’t know you have

**DOI:** 10.1038/s44271-023-00023-y

**Published:** 2023-09-21

**Authors:** Antonia Eisenkoeck

**Affiliations:** Communications Psychology, https://www.nature.com/commspsychol

## Abstract

A recent study in *Cognition* provides evidence for phenomenal consciousness without knowledge thereof, by virtue of a sound detection and discrimination paradigm.

**Figure:**
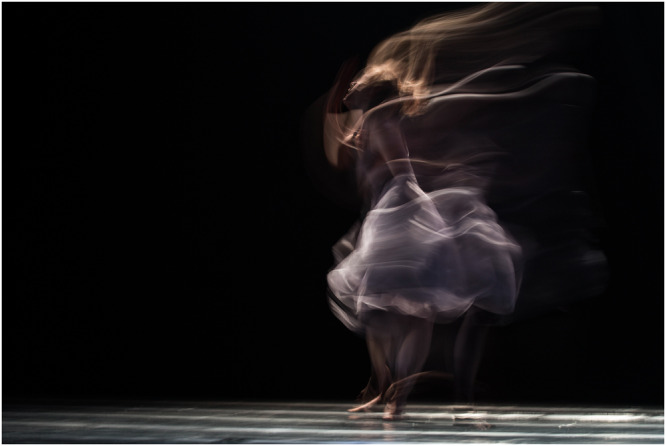
Ahmad Odeh on unsplash.com

Can one have a conscious experience without knowing it? Whether consciousness without access to it exists has been widely debated. At the heart of it lies the question whether the distinction between phenomenal consciousness—what it is like to have an experience—and access consciousness—the ability to report it—is a purely conceptual idea or indeed a practical possibility.

The key challenge to tackling this question experimentally is that participants’ report of experience inevitably implies access to it. In other words, paradigms used in consciousness research typically study situations in which phenomenal experience and access to it are intertwined.

Yoni Amir and colleagues developed a new paradigm to circumvent this problem: Participants were presented with a range of sounds, sometimes including pink noise. The sounds were gradually turned off until only the pink noise remained. If people denied hearing the pink noise but noticed a change when it then suddenly stopped, according to the researchers, this showed experience without online access to it^1^. When subsequently asked to determine which out of two pink noise signals was the one they had unknowingly been presented with, people performed better than chance to identify the target sound—which authors argue is evidence for phenomenal (i.e., qualitative) experience. As evidence that this effect is not driven by unconscious processing (or at least not entirely), Amir et al. show that participants performed significantly worse (and at chance) in trials in which they had not noticed the abrupt end of the pink noise, in other words, in trials in which participants had had no experience of the sound.

Taken together, this study not only provides some compelling evidence that experience without access is not only a conceptual but also a practical possibility, but also offers a promising paradigm that can be used to continue on this scientific quest.
